# Supplementary material for: Mechanical control of neural plate folding by apical domain alteration
Source: Nat Commun. 2023 Dec 20;14:8475. doi: 10.1038/s41467-023-43973-x (PMC10733383; doi:10.1038/s41467-023-43973-x)
Supplement: Supplementary file 1 — Supplementary Information [file 41467_2023_43973_MOESM1_ESM.pdf]

Mechanical control of neural plate folding by apical domain alteration

Miho Matsuda [1], Jan Rozman [2], Sassan Ostvar [3], Karen E. Kasza [3] and Sergei Y. Sokol [1]

Supplementary information: Figures S1-S11, Table S1, Movies 1-5.

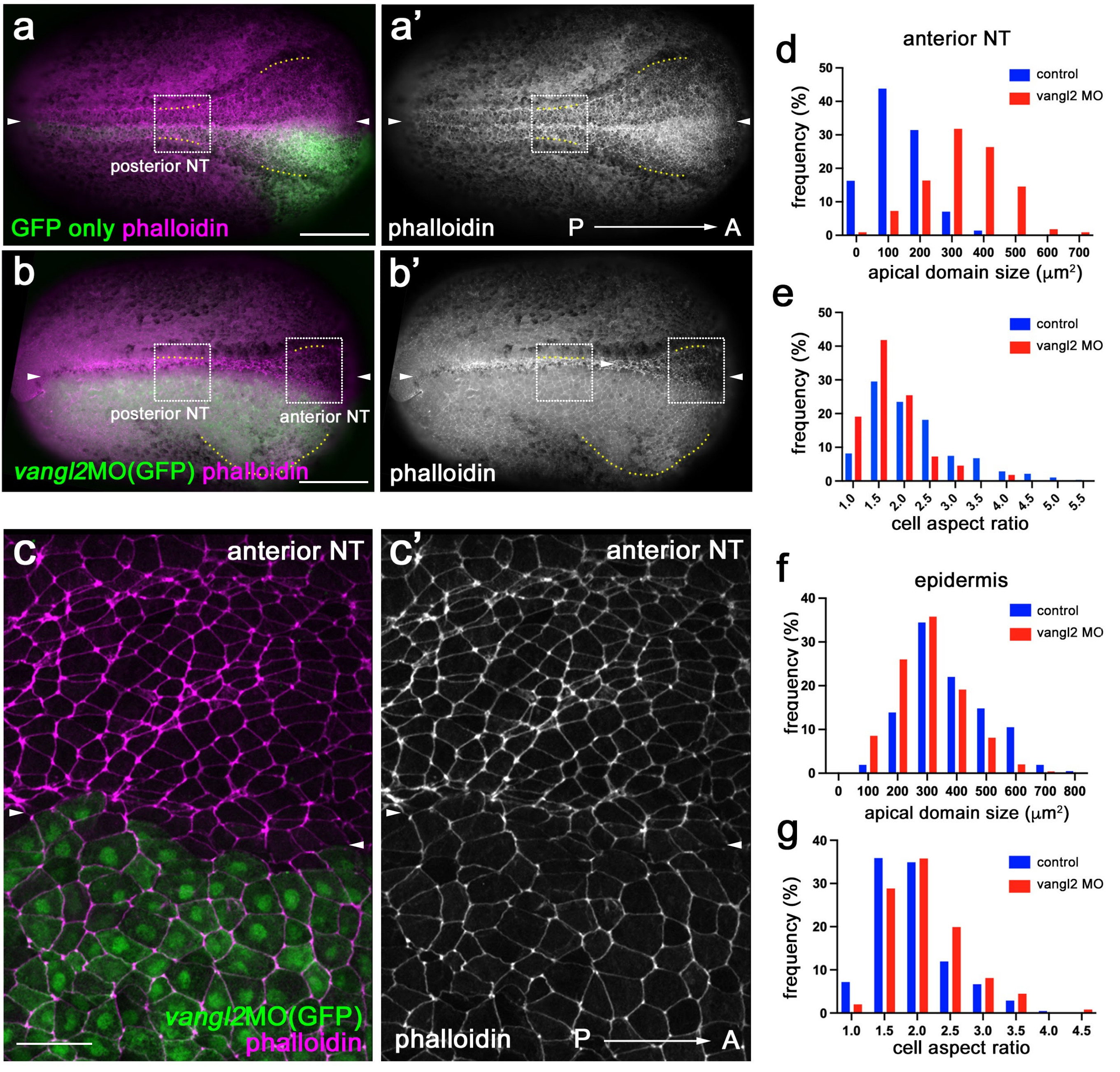
a
posterior NT
GFP only phalloidin
a'
phalloidin
P
A
b
posterior NT
anterior NT
vangl2MO(GFP) phalloidin
b'
phalloidin
c
anterior NT
vangl2MO(GFP)
phalloidin
c'
anterior NT
phalloidin
P
A
d
anterior NT
control
vangl2 MO
frequency (%)
apical domain size (μm²)
e
control
vangl2 MO
frequency (%)
cell aspect ratio
f
epidermis
control
vangl2 MO
frequency (%)
apical domain size (μm²)
g
control
vangl2 MO
frequency (%)
cell aspect ratio

Supplementary Figure 1. PCP-dependent cell heterogeneity in the anterior and posterior neural plate.

(a-b') Representative images of injected embryos. GFP RNA and *vangl2* MO or control GFP RNA was injected into one dorsal blastomere of 4-cell stage embryos. Embryos at stage 15-16 were stained with phalloidin. The approximate locations of posterior and anterior NP images in Figs. 2a-b' and S1c-c' and are indicated in white boxes. The midline is indicated by white arrowheads. (c, c') *vangl2* knockdown increased apical domain size and decreased cell aspect ratio in the anterior neural plate. Scale bars are 100 μm in a, b and 50 μm in c. Experiments were repeated three times. (d) Comparison of apical domain size in *vangl2* MO-injected and control cells. See Methods. n=283 cells for control. N=110 cells for *vangl2* MO. (e) Cell aspect ratio for cells in c. (f) Apical domain size in the *vangl2* MO-injected and control epidermis. Note that epidermis in *vangl2* MO has smaller apical domains than control epidermis. (g) Cell aspect ratio in the *vangl2* MO injected and control epidermal cells. n=209, for control cells from 6 embryos. n=246, for *vangl2* MO containing cells from 9 embryos. Experiments were repeated three times. Statistical significance was assessed using the Kolmogorov-Smirnov test. $p<0.0000000001$ (d). $p=0.000000939$ (e). $p=0.000002587$ (f). $p=0.0027$ (g).

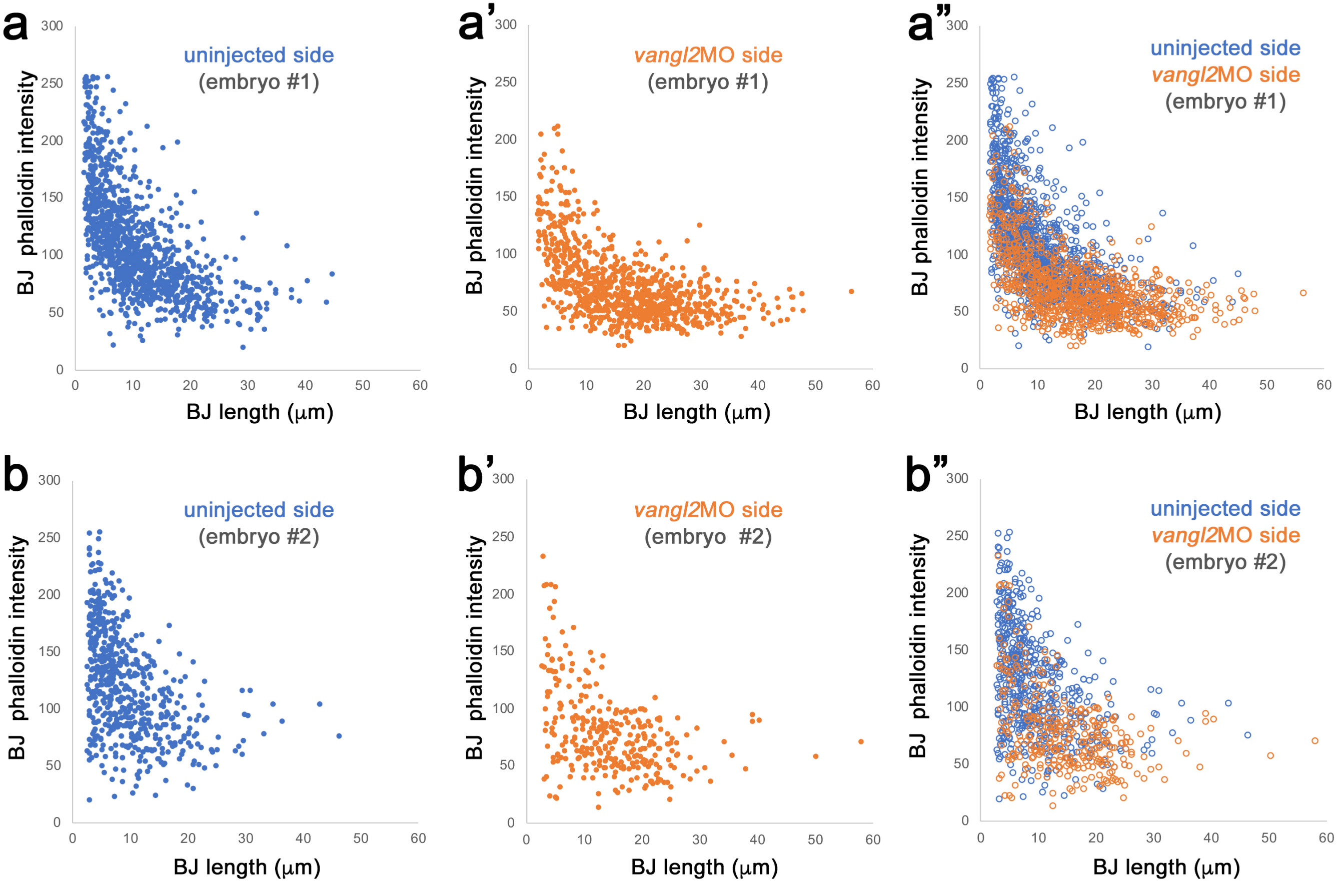
a
uninjected side
(embryo #1)
BJ phalloidin intensity
BJ length (μm)
a'
vangl2MO side
(embryo #1)
a"
uninjected side
vangl2MO side
(embryo #1)
b
uninjected side
(embryo #2)
b'
vangl2MO side
(embryo #2)
b"
uninjected side
vangl2MO side
(embryo #2)

Supplementary Figure 2. Quantification of bicellular junction length and phalloidin intensity in Vangl2-depleted and wild-type neuroepithelial cells.

(a-b”) Quantification of bicellular junction (BJ) length and the average relative phalloidin intensity in the area bordering the anterior and the posterior regions of NP. Two different embryos are shown in a and b. The uninjected control side (blue in a, a”, b, b”). *vangl2* MO-injected side (orange in a’, a”, b’, b”). Individual dots represent individual BJs. n=1121 cells for uninjected in a, a”. n=856 cells for *vang2* MO in a’, a”. n=571 cells for uninjected in b, b”, n=307 cells for *vangl2* MO in b’, b”. BJs were used for quantification to avoid the complexity of tricellular junction regulation.

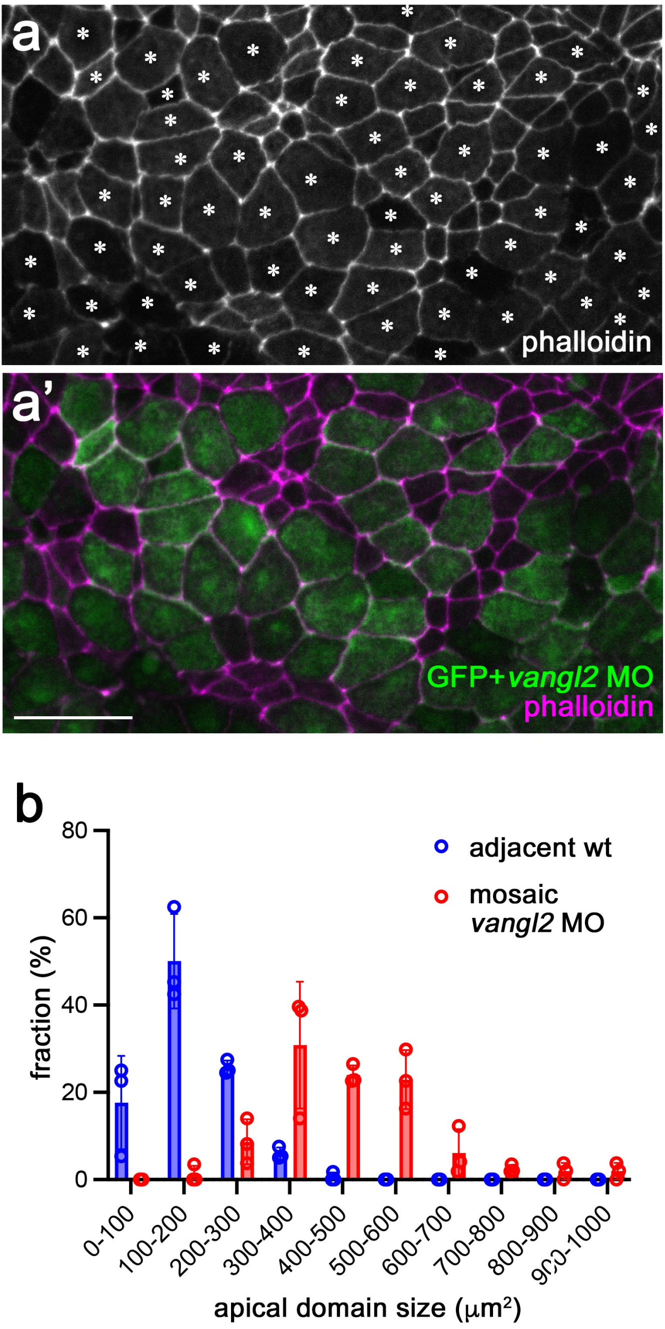
a
phalloidin
a'
GFP+vangl2 MO
phalloidin
b
80
60
40
20
0
fraction (%)
adjacent wt
mosaic vangl2 MO
0-100
100-200
200-300
300-400
400-500
500-600
600-700
700-800
800-900
900-1000
apical domain size (μm²)

Supplementary Figure 3. Effects of Vangl2 depletion on apical domain size.

Embryos were injected at the 8-16 cell stage with *vangl2* MO to achieve mosaic depletion. (a) Representative image of phalloidin-stained anterior neuroectoderm with mosaic *vangl2* depleted cells (asterisks in a, green in a'). GFP was co-injected with *vangl2* MO as a tracer. (b) Quantification of apical domain size in mosaic *vangl2* MO cells (red) and the adjacent wild-type control cells (blue). n=162 for *vangl2* MO cells and n=151 for adjacent wile-type cells from three embryos. som: somite, nt: notochord, ne: neuroectoderm, n-ne: non-neuroectoderm. The Kolmogorov-Smirnov test. $p<0.0000000001$. Scale bar: 20 μm.

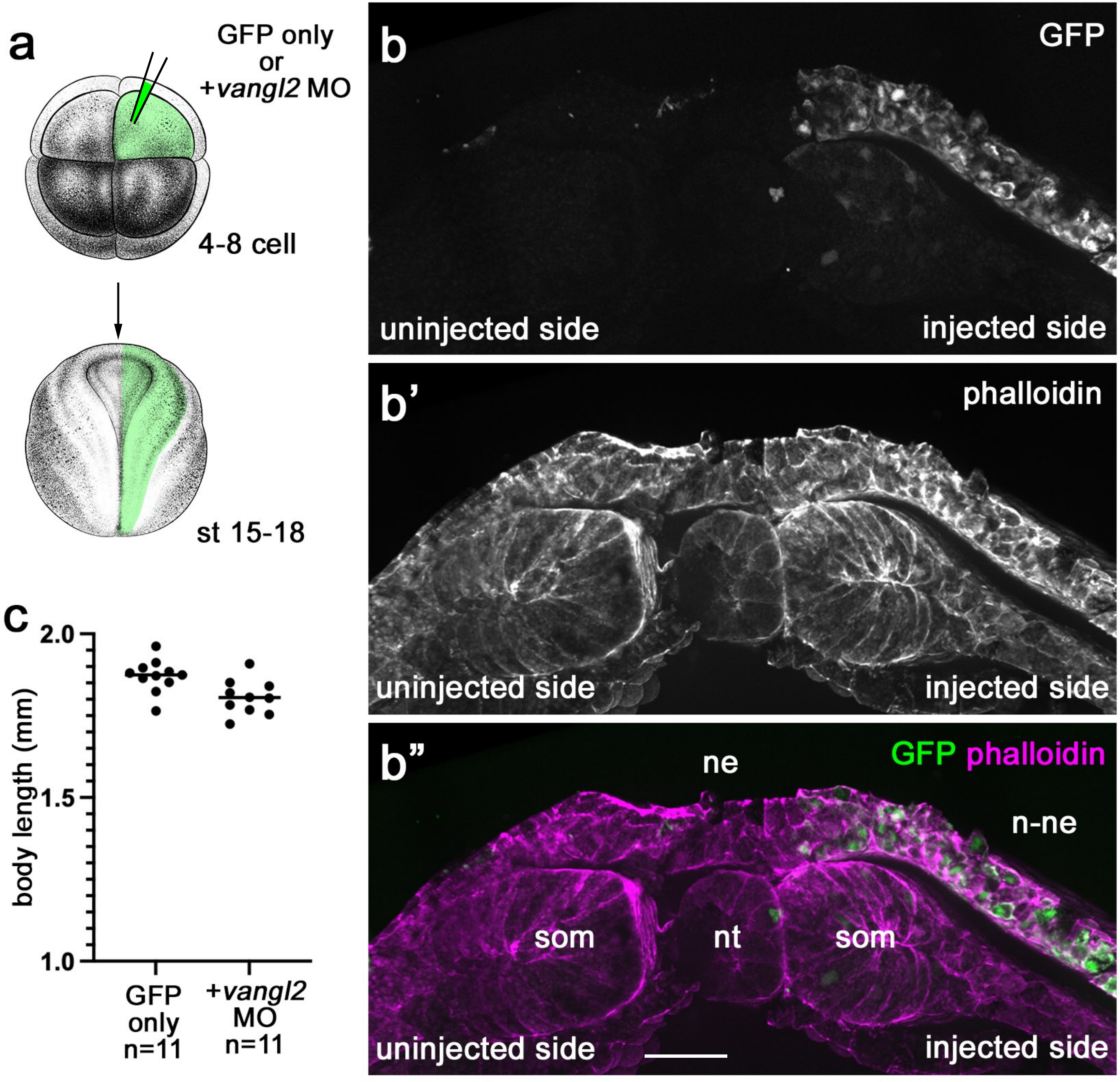
a
GFP only
or
+vangl2 MO
4-8 cell
st 15-18
b
GFP
uninjected side
injected side
b'
phalloidin
uninjected side
injected side
b"
GFP phalloidin
ne
n-ne
som
nt
som
uninjected side
injected side
c
2.0
1.5
1.0
body length (mm)
GFP only n=11
+vangl2 MO n=11

Supplementary Figure 4. Targeting of neuroectoderm by microinjection and the quantification of the body length in mosaic Vangl2 morphants.

(a) Schematics of the injection experiments. Adapted from *Xenopus* illustrations © Natalya Zahn (2022), Xenbase (www.xenbase.org RRID:SCR_003280)[1]. (b-b”) Representative cross-section of phalloidin-stained stage 15 embryo (dorsal region) that was injected in a dorsal animal blastomere at the 4-8-cell stage with 50 pg of GFP RNA as a lineage tracer. GFP fluorescence is imaged directly after cryosectioning. b and b’, separate channels, b”, merged channels. Experiments were repeated three times. Scale bar: 50 μm. (c) Quantification of the body length of mosaic *vangl2* morphants at stages 16-17. n=11, for control GFP expressing embryos. n=11, for *vangl2* MO+GFP embryos. The Student’s t-test. Two-sided. p=0.0089.

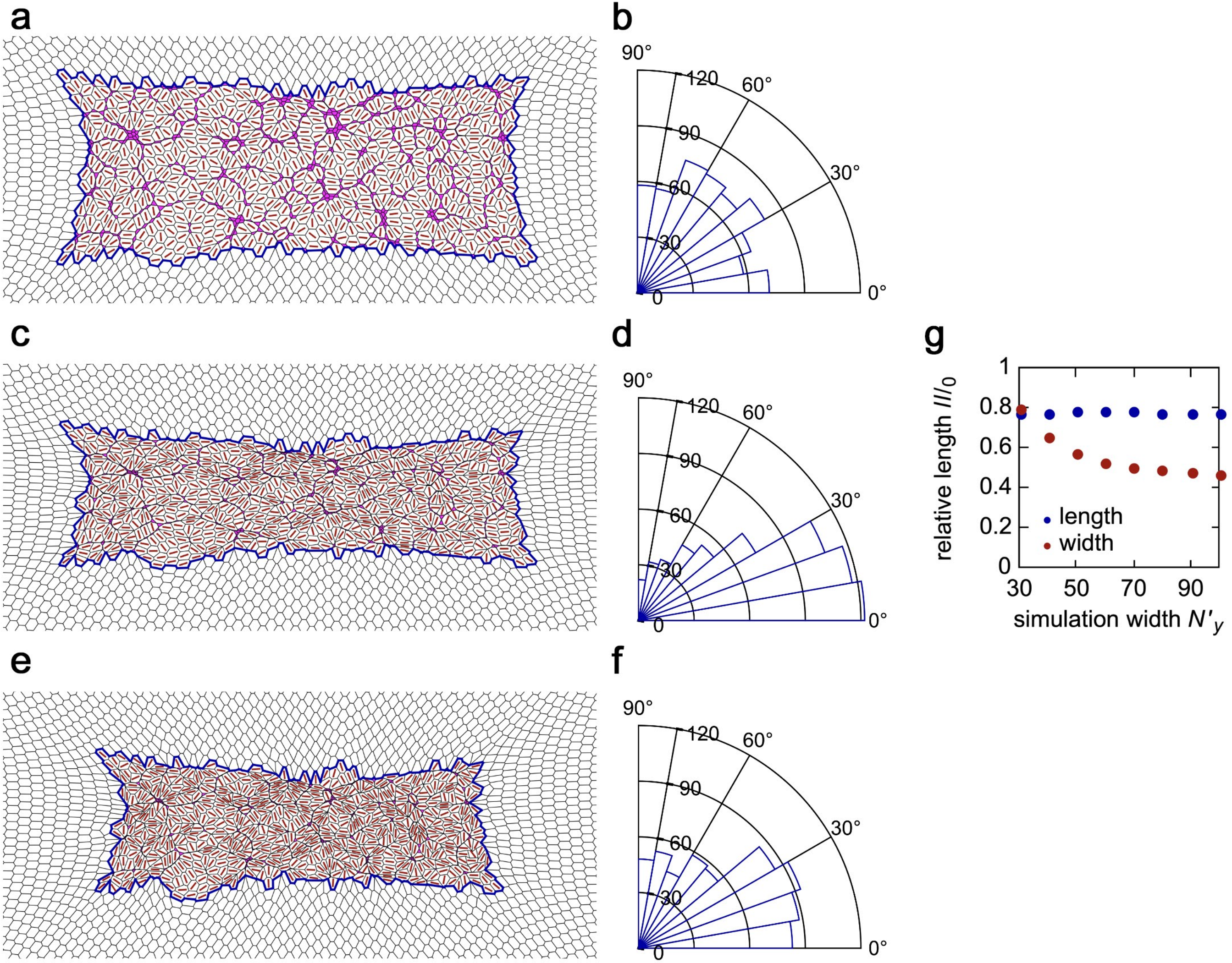

a
b
90°
120
60°
90
30°
60
30
0
0°
c
d
g
relative length l/l_0
1
0.8
0.6
0.4
0.2
0
length
width
30
50
70
90
simulation width N'_y
e
f

Supplementary Figure 5. Effect of the external region on cell alignment in simulations.

(a, b) Model tissue after relaxation (a) and distribution of angles between the AP axis (horizontal line) and cell elongation direction (b) at $t = 2000$ for $N'_x = 100$ and $N'_y = 34$. (c, d) Model tissue after relaxation (c) and distribution of angles between the AP axis and cell elongation direction (D) at $t = 2000$ for $N'_x = 100$ and $N'_y = 100$. (e, f) Model tissue after relaxation (e) and distribution of angles between the AP axis and cell elongation direction (f) at $t = 20000$ for $N'_x = 180$ and $N'_y = 180$. (g) Central length (along the AP axis) and width (perpendicular to the AP axis) of the model tissue, relative to those lengths at the start of the simulation, as a function of N'_y at $t = 2000$, keeping $N'_x = 100$.

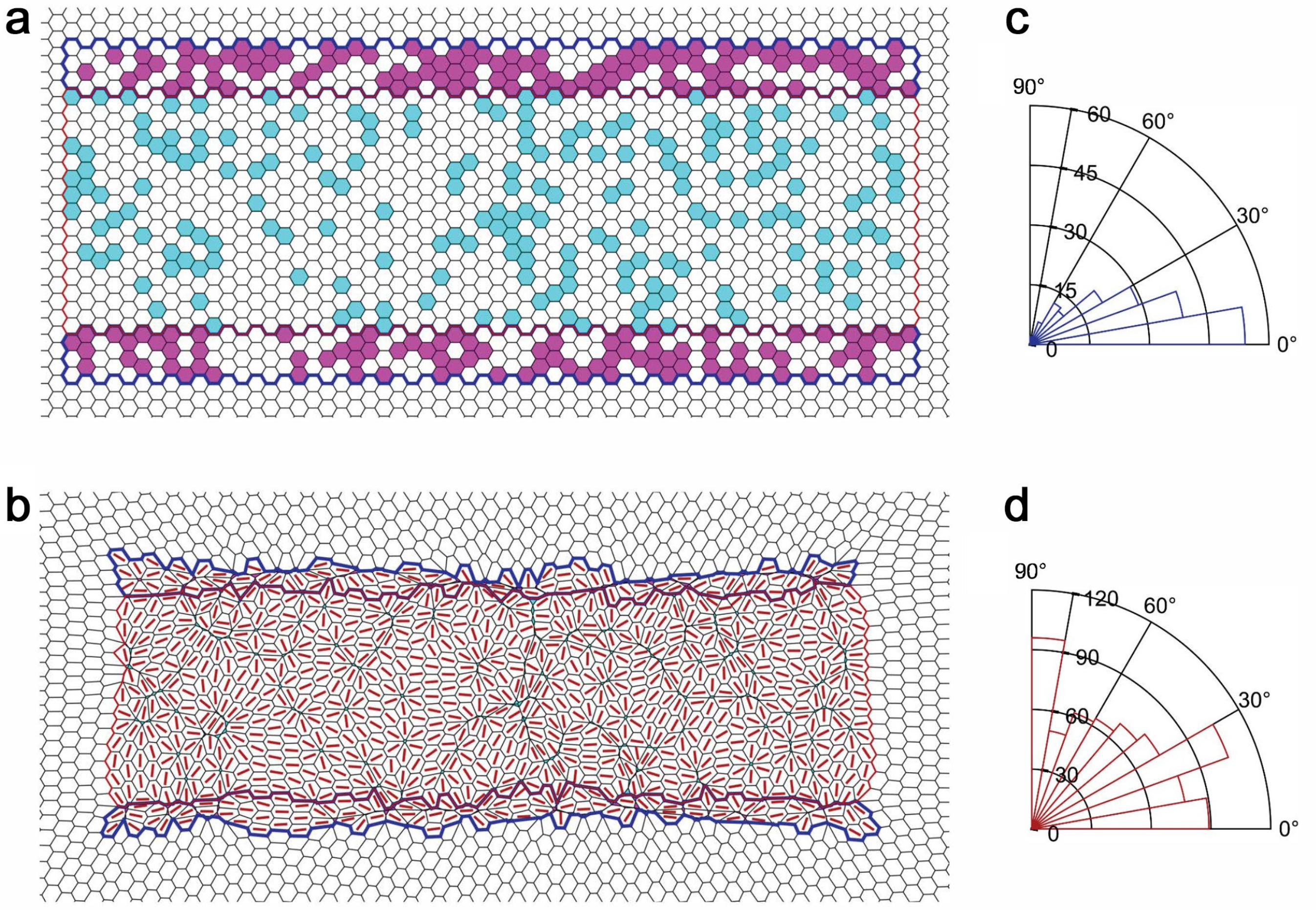
a
b
c
90°
60
60°
45
30
30°
15
0
0°
d
90°
120
60°
90
30°
60
30
0
0°

Supplementary Figure 6. Apically constricting 'hinge cells' contribute to neural plate cell morphology and orientation.

(a) Zoom on the neural plate region of the vertex model initial condition with separate dorsolateral hinge regions. Blue border outlines the hinges, whereas a red border outlines the region between them. Constricting cells are shown in magenta for the hinges and in cyan for the remainder of the model posterior neural plate (tissue shown for $P_h = 0.5$ and $P_c = 0.2$). (b) Model tissue from panel a after relaxation at $t = 2000$. Red lines show the direction of cell elongation. (c, d) Distribution of angles between the AP axis (horizontal line) and cell elongation direction for the model tissue in panel b for non-constricting cells in the hinges (c) and in the remainder of the neural plate (d).

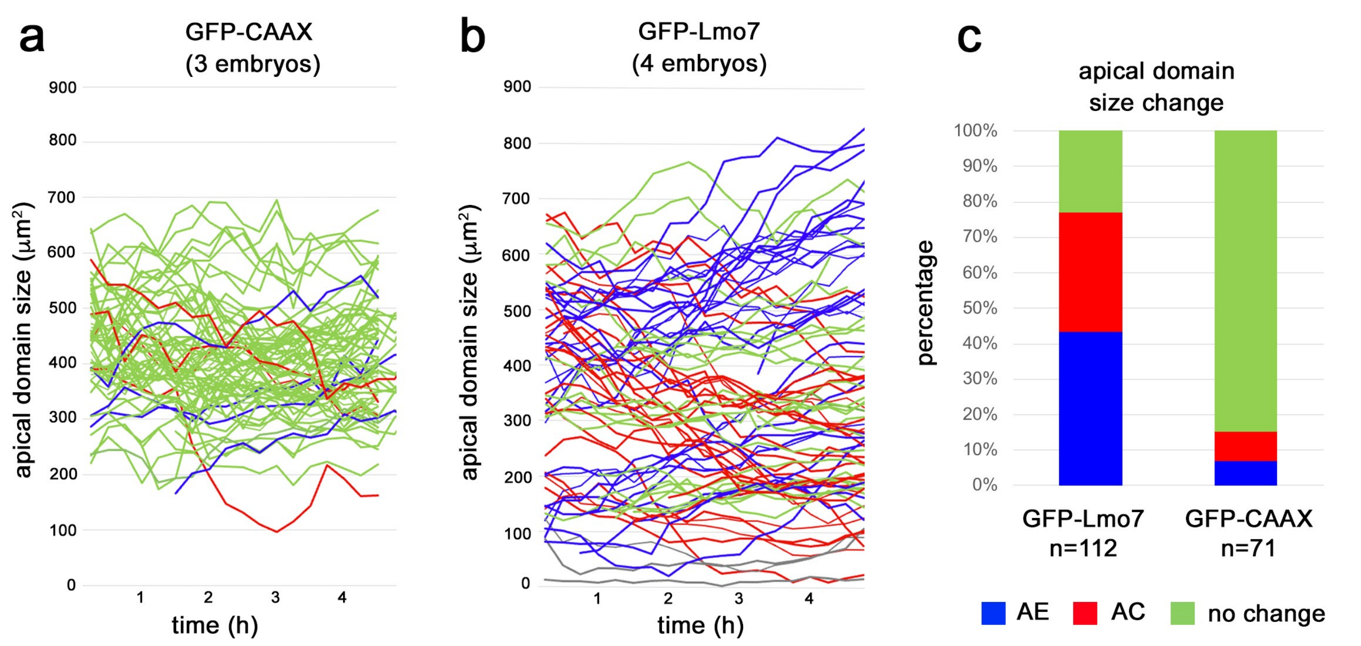
a
GFP-CAAX
(3 embryos)
apical domain size (μm²)
time (h)
b
GFP-Lmo7
(4 embryos)
apical domain size (μm²)
time (h)
c
apical domain
size change
percentage
GFP-Lmo7
n=112
GFP-CAAX
n=71
AE
AC
no change

Supplementary Figure 7. Heterogeneous response of ectodermal cells to Lmo7.

(a, b) Quantification of apical domain size dynamics in GFP-CAAX (a) or GFP-Lmo7 (b) embryos. GFP-CAAX or Lmo7 RNA was injected into four animal blastomeres of 4-8-cell embryos as shown in Fig. 5a. Each line represents apical domain size change of an individual cell over 4-5 hours. AC (red) and AE (blue) cells show more than 20% decrease or increase in their apical domain size, respectively. Cells with less than 20% changes ('no change') are shown in green. Grey lines indicate cells which had the apical domain smaller than 150 μm^2 at the beginning of time-lapse imaging and remained small. Data for 71 GFP-CAAX cells are from 3 embryos. Data for 112 GFP-Lmo7 cells are from 4 embryos. (c) Percentage of AC, AE and 'no change' cells in a and b. Repeated at least three times. The Freeman-Halton extension of Fisher's exact test. p= 2.081 E-16.

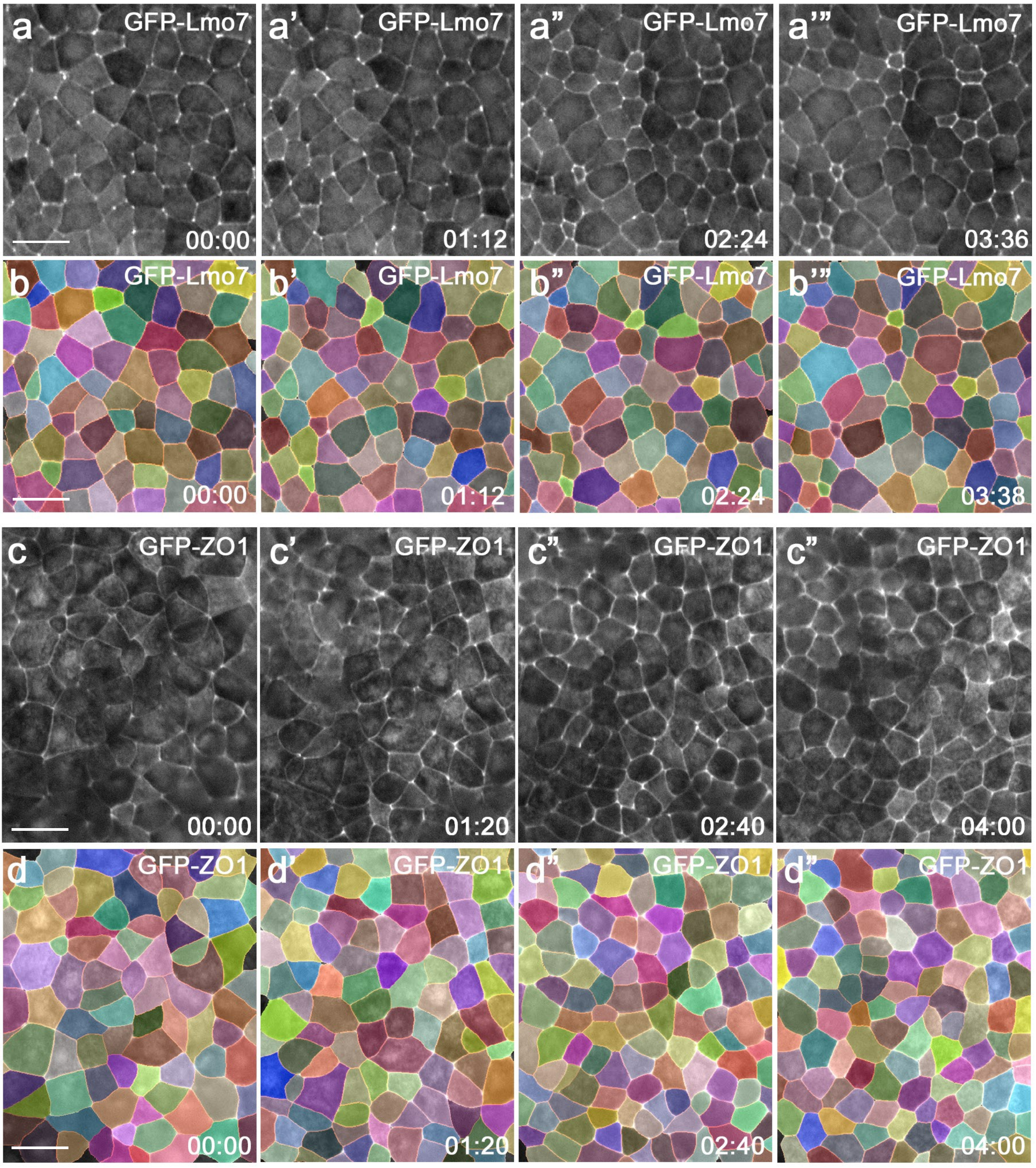

a
GFP-Lmo7
00:00
a’
GFP-Lmo7
01:12
a”
GFP-Lmo7
02:24
a’”
GFP-Lmo7
03:36
b
GFP-Lmo7
00:00
b’
GFP-Lmo7
01:12
b”
GFP-Lmo7
02:24
b’”
GFP-Lmo7
03:38
c
GFP-ZO1
00:00
c’
GFP-ZO1
01:20
c”
GFP-ZO1
02:40
c”
GFP-ZO1
04:00
d
GFP-ZO1
00:00
d’
GFP-ZO1
01:20
d”
GFP-ZO1
02:40
d”
GFP-ZO1
04:00

Supplementary Figure 8. Cell segmentation and cell tracking in Lmo7-expressing and control ectoderm.

Representative still images of stage 11-12 ectoderm expressing GFP-Lmo7 (a-a’’’) and GFP-ZO1 (c-c’’’) from time-lapse imaging. Segmented images of a-a’’’ and c-c’’’ are shown in b-b’’’ and d-d’’’, respectively. See Movie 2 and Movie 3. For details of segmentation and cell tracking, see Methods. Experiments were repeated at least three times. Scale bars are 40 μm.

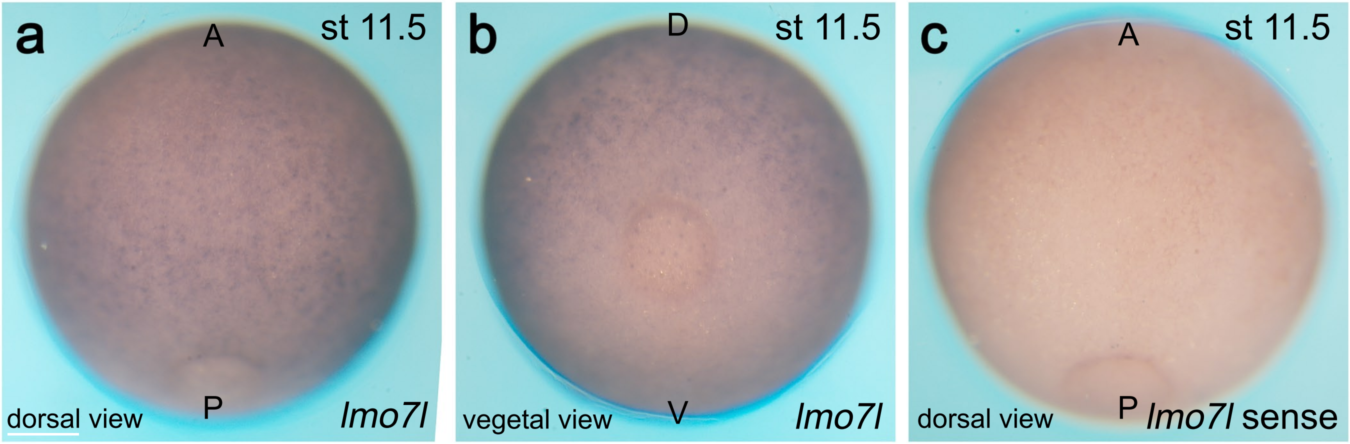
a
st 11.5
A
P
dorsal view
lmo7l
b
st 11.5
D
V
vegetal view
lmo7l
c
st 11.5
A
P
dorsal view
lmo7l sense

Supplementary Figure 9. Non-homogeneous distribution of *lmo7l* transcripts in late gastrulae.

Whole mount *in situ* hybridization was carried out with *lmo7l* antisense and sense probes using stage 11.5 embryos. Representative embryo images are shown. (a,b) *lmo7l* antisense probe. Dorsal view (a). Vegetal view (b). (c) *lmo7* sense probe. Dorsal view. A: anterior. P: posterior. Scale bar: 200 μm.

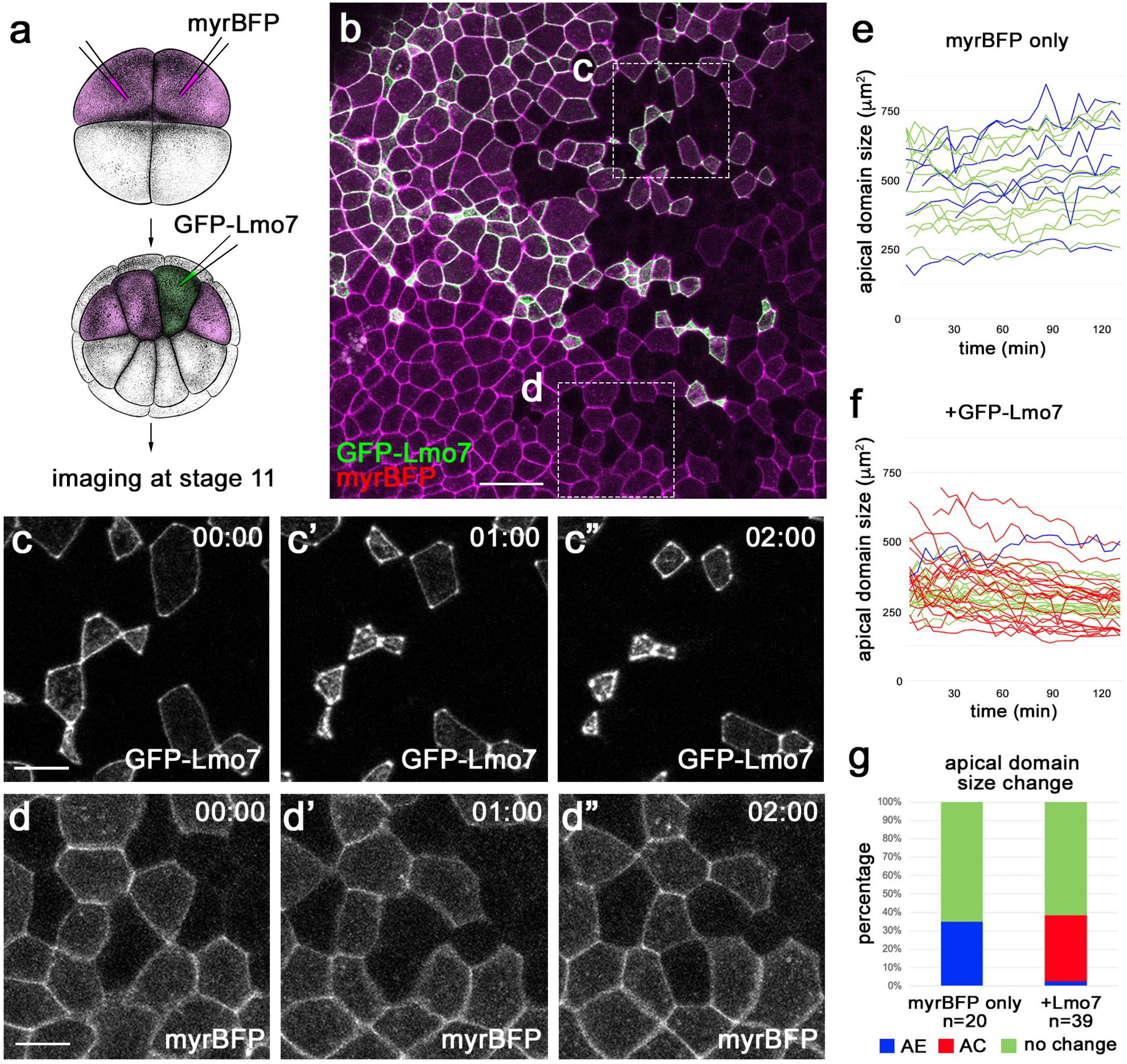
a
myrBFP
GFP-Lmo7
imaging at stage 11
b
c
d
GFP-Lmo7
myrBFP
e
myrBFP only
apical domain size (μm2)
time (min)
f
+GFP-Lmo7
g
apical domain size change
percentage
myrBFP only n=20
+Lmo7 n=39
AE
AC
no change
c 00:00
c' 01:00
c" 02:00
GFP-Lmo7
d 00:00
d' 01:00
d" 02:00
myrBFP

Supplementary Figure 10. Apical constriction of mosaically expressing Lmo7 cells.

(a) Scheme of the experiment. myrBFP RNA (50 pg) was injected into two ventral blastomeres of 4-cell stage embryos. GFP-Lmo7 RNA (100 pg) was subsequently injected into one ventral blastomere of 16-cell stage embryos. The animal ectoderm was imaged at stage 11. Adapted from *Xenopus* illustrations © Natalya Zahn (2022), Xenbase (www.xenbase.org RRID:SCR_003280)[1]. (b) Representative image of myrBFP and GFP-Lmo7 expressing embryos. (c-d") Representative still images of GFP-Lmo7 (c-c") and myrBFP (d-d") cells from time-lapse imaging. (e, f) Quantification of apical domain size dynamics in myrBFP (e) and GFP-Lmo7 (f) cells. Each line represents apical domain size changes of individual cells over 2 hrs. n=20 cells for myrBFP only and n=39 for myrBFP+GFP-Lmo7 from three embryos. (g) Percentage of AC (red), AE (blue) and 'no change' (green) cells (See Fig. 5 legend) is shown in e and f. the Freeman-Halton extension of Fisher's exact test. p= 0.000065. The data represents 3-5 independent experiments.

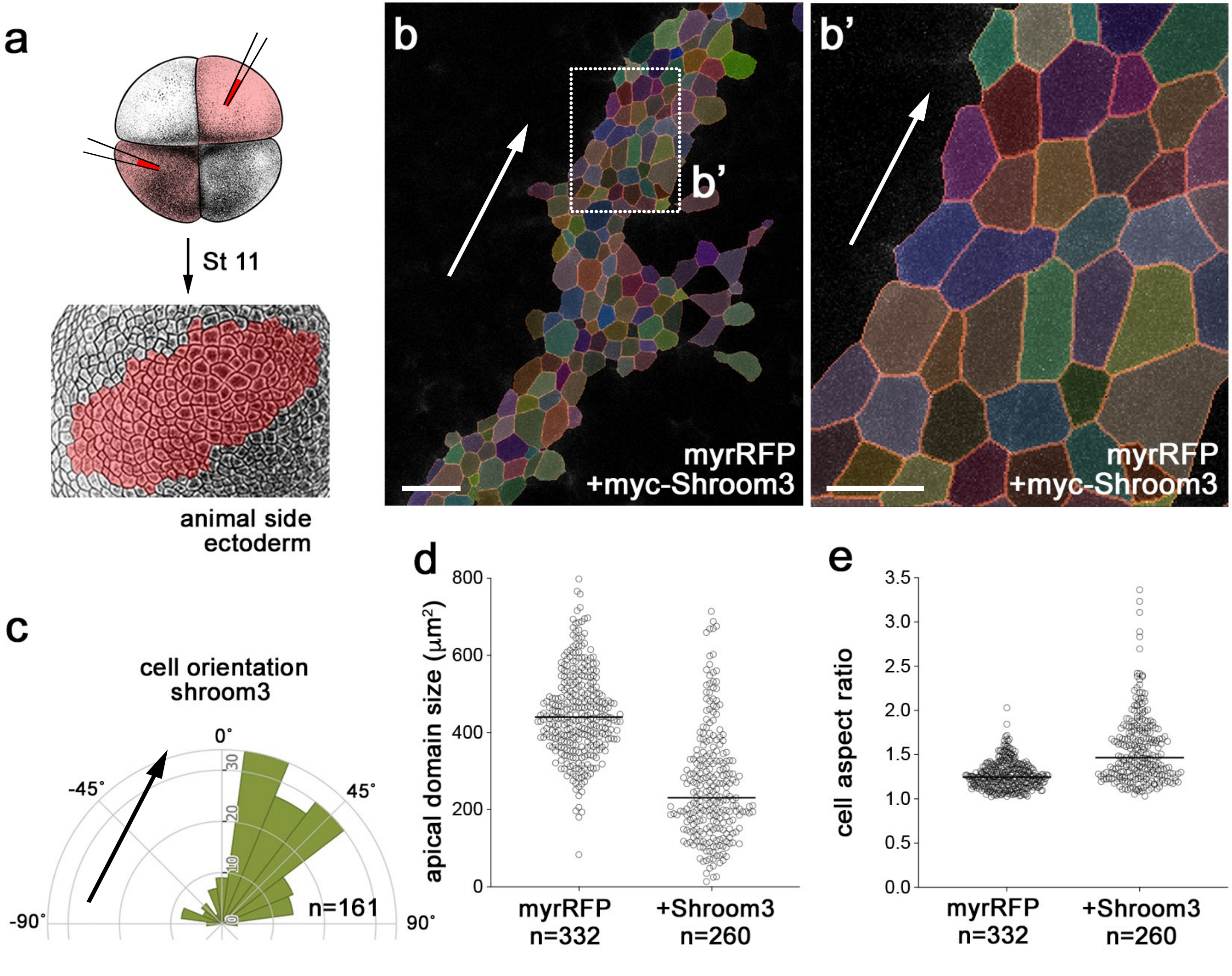

a
St 11
animal side ectoderm
b
b'
myrRFP +myc-Shroom3
b'
myrRFP +myc-Shroom3
c
cell orientation shroom3
0°
-45°
45°
-90°
90°
n=161
d
apical domain size (μm²)
800
600
400
200
0
myrRFP n=332
+Shroom3 n=260
e
cell aspect ratio
3.5
3.0
2.5
2.0
1.5
1.0
0.5
0.0
myrRFP n=332
+Shroom3 n=260

Supplementary Figure 11. Shroom3 promotes apical domain heterogeneity.

(a) Scheme of the experiment for b-e. myc-Shroom3 (40 pg) and myrRFP (50 pg) RNAs were co-injected into two opposing animal blastomeres of 4-8 cell stage embryos. Animal pole ectoderm was imaged at stage 11. Adapted from *Xenopus* illustrations © Natalya Zahn (2022), Xenbase (www.xenbase.org RRID:SCR_003280)[1]. (b, b') Representative segmented Shroom3-expressing ectoderm. Rectangular area in b is enlarged in b'. (c) Rose plot depicting cell orientation of Shroom3 cells in b and b'. n=161 cells from one embryo. (c, e) Quantification of apical domain size (d) and cell aspect ratio (e) of myrRFP cells and myrRFP+Shroom3 cells. n=332 cells for myrRFP and n=260 cells (myrRFP+Shroom3). Two embryos each. The Kolmogorov-Smirnov test. $p<0.000000001$ (d). $p<0.000000001$ (e). Experiments were repeated three times. Scale bars are 50 μm in b and 20 μm in b'.

Supplementary References

1. Zahn et al., Normal Table of Xenopus development: a new graphical resource. Development (2022) 149 (14): dev200356. https://doi.org/10.1242/dev.200356
