# Supplementary material for: Mechanical control of neural plate folding by apical domain alteration
Source: Nat Commun. 2023 Dec 20;14:8475. doi: 10.1038/s41467-023-43973-x (PMC10733383; doi:10.1038/s41467-023-43973-x)
Supplement: Supplementary file 3 — Description of Additional Supplementary Files [file 41467_2023_43973_MOESM3_ESM.pdf]

Description of Additional Supplementary Files

Supplementary Movie 1. Ectoderm morphology in embryos expressing GFP-Lmo7.

Time-lapse recording of GFP-Lmo7 expressing cells in stage 11 *Xenopus* ectoderm. 1 ng of GFP-Lmo7 RNA was injected into the two animal blastomeres of 4-cell stage embryos. Animal side view. Images were taken every 10 min on the Zeiss AxioZoom stereo microscope. Duration is 3 hrs and 20 min.

Supplementary Movie 2. Apical domain dynamics of GFP-Lmo7 cells.

Time-lapse recording of GFP-Lmo7 expressing cells in stage 11 *Xenopus* ectoderm. 100 pg of GFP-Lmo7 RNA was injected into four animal blastomeres of 4-cell stage embryos to obtain uniform expression. Animal view. Images were taken every 10 min on the Zeiss AxioZoom stereo microscope. Duration of the time-lapse video is 4 hrs.

Supplementary Movie 3. Apical domain dynamics of embryonic ectoderm cells expressing ZO1.

Time-lapse recording of control 3xGFP-ZO1 expressing cells in stage 11 *Xenopus* ectoderm. 150 pg of 3xGFP-ZO1 RNA was injected into four animal blastomeres of 4-cell stage embryos to obtain uniform expression. Animal view. Images were taken every 8 min on the Zeiss AxioZoom stereo microscope. Duration of the time-lapse video is 3 hrs and 36 min.

Supplementary Movie 4. Apical domain dynamics of embryonic ectoderm cells uniformly expressing Lmo7.

Time-lapse recording of cells co-expressing 3xGFP-ZO1 and Flag-Lmo7 in stage 11 *Xenopus* ectoderm. 100 pg of 3xGFPZO1 and 100 pg of Flag-Lmo7 RNA was injected into four animal blastomeres of 4-cell stage embryos to obtain uniform “sheet” expression. Animal view. Images were taken every 6 min on the Zeiss LSM880 confocal microscope. Duration is 1 hr and 42 min.

Supplementary Movie 5. Apical domain dynamics of ectoderm in embryos mosaically expressing Lmo7.

Time-lapse recording of cells co-expressing 3xGFP-ZO1 and Flag-Lmo7 in stage 11 *Xenopus* ectoderm. 100 pg of 3xGFPZO1 and 100 pg of Flag-Lmo7 RNA were injected into one animal blastomere of 4-cell stage embryos to obtain “mosaic” expression. Animal view. Images were taken every 6 min on the Zeiss LSM880 confocal microscope system. Duration is 1 hr 54 min.
