# Supplementary material for: Mechanical control of neural plate folding by apical domain alteration
Source: Nat Commun. 2023 Dec 20;14:8475. doi: 10.1038/s41467-023-43973-x (PMC10733383; doi:10.1038/s41467-023-43973-x)
Supplement: Supplementary file 9 — Reporting Summary [file 41467_2023_43973_MOESM9_ESM.pdf]

nature portfolio

Corresponding author(s): Sergei Sokol

Last updated by author(s): Nov 6, 2023

Reporting Summary

Nature Portfolio wishes to improve the reproducibility of the work that we publish. This form provides structure for consistency and transparency in reporting. For further information on Nature Portfolio policies, see our Editorial Policies and the Editorial Policy Checklist.

Statistics

For all statistical analyses, confirm that the following items are present in the figure legend, table legend, main text, or Methods section.

n/a	Confirmed	
☐	☒	The exact sample size (*n*) for each experimental group/condition, given as a discrete number and unit of measurement
☐	☒	A statement on whether measurements were taken from distinct samples or whether the same sample was measured repeatedly
☐	☒	The statistical test(s) used AND whether they are one- or two-sided<br>*Only common tests should be described solely by name; describe more complex techniques in the Methods section.*
☒	☐	A description of all covariates tested
☒	☐	A description of any assumptions or corrections, such as tests of normality and adjustment for multiple comparisons
☐	☒	A full description of the statistical parameters including central tendency (e.g. means) or other basic estimates (e.g. regression coefficient) AND variation (e.g. standard deviation) or associated estimates of uncertainty (e.g. confidence intervals)
☒	☐	For null hypothesis testing, the test statistic (e.g. *F*, *t*, *r*) with confidence intervals, effect sizes, degrees of freedom and *P* value noted<br>*Give P values as exact values whenever suitable.*
☒	☐	For Bayesian analysis, information on the choice of priors and Markov chain Monte Carlo settings
☒	☐	For hierarchical and complex designs, identification of the appropriate level for tests and full reporting of outcomes
☒	☐	Estimates of effect sizes (e.g. Cohen's *d*, Pearson's *r*), indicating how they were calculated

Our web collection on statistics for biologists contains articles on many of the points above.

Software and code

Policy information about availability of computer code

Data collection	Zeiss Zen (blue edition) Ver 3.7.97.04000. Andor Fusion, Ver 2.
Data analysis	ImageJ2 Ver 2.9.0/1.54g. GraphPad Prism. Ver 10.1.0. Python package Cellpose v2.0.5. Python package BayesianTracker (btrack v0.4.5). scikit-image v0.21.0. sknw. Wolfram Mathematica (Versions 12.1 and 13.2).<br>C++ (-std=c++14). Custom code used in this study to analyze protein localization patterns and tissue structure is available at https://github.com/kaszalab/xenopus-protein-localization

For manuscripts utilizing custom algorithms or software that are central to the research but not yet described in published literature, software must be made available to editors and reviewers. We strongly encourage code deposition in a community repository (e.g. GitHub). See the Nature Portfolio guidelines for submitting code & software for further information.

Data

Policy information about availability of data

All manuscripts must include a data availability statement. This statement should provide the following information, where applicable:

- Accession codes, unique identifiers, or web links for publicly available datasets
- A description of any restrictions on data availability
- For clinical datasets or third party data, please ensure that the statement adheres to our policy

All data supporting the findings of this study are available within the paper, its supplementary information and source data file provided with this paper.

Research involving human participants, their data, or biological material

Policy information about studies with human participants or human data. See also policy information about sex, gender (identity/presentation), and sexual orientation and race, ethnicity and racism.

Reporting on sex and gender	n/a
Reporting on race, ethnicity, or other socially relevant groupings	n/a
Population characteristics	n/a
Recruitment	n/a
Ethics oversight	n/a

Note that full information on the approval of the study protocol must also be provided in the manuscript.

Field-specific reporting

Please select the one below that is the best fit for your research. If you are not sure, read the appropriate sections before making your selection.

☒ Life sciences ☐ Behavioural & social sciences ☐ Ecological, evolutionary & environmental sciences

For a reference copy of the document with all sections, see nature.com/documents/nr-reporting-summary-flat.pdf

Life sciences study design

All studies must disclose on these points even when the disclosure is negative.

Sample size	No sample-size calculation was performed.
Data exclusions	No data was excluded from the analyses.
Replication	The experimental findings were confirmed by at least three independent replication.
Randomization	In each attempt of experiment, embryos obtained from the same pair of female and male frogs were randomly divided into control and experimental groups.
Blinding	Blinding is not relevant to this study.

Reporting for specific materials, systems and methods

We require information from authors about some types of materials, experimental systems and methods used in many studies. Here, indicate whether each material, system or method listed is relevant to your study. If you are not sure if a list item applies to your research, read the appropriate section before selecting a response.

Materials & experimental systems

n/a	Involved in the study
☒	☐ Antibodies
☒	☐ Eukaryotic cell lines
☒	☐ Palaeontology and archaeology
☐	☒ Animals and other organisms
☒	☐ Clinical data
☒	☐ Dual use research of concern
☒	☐ Plants

Methods

n/a	Involved in the study
☒	☐ ChIP-seq
☒	☐ Flow cytometry
☒	☐ MRI-based neuroimaging

Animals and other research organisms

Policy information about studies involving animals; ARRIVE guidelines recommended for reporting animal research, and Sex and Gender in Research

Laboratory animals	Wild-type Xenopus laevis were purchased from commercial vendors, Nasco and Xenopus1. 1-3 years old. No specific strains were defined in Xenopus laevis.
Wild animals	The study does not involve wild animals.
Reporting on sex	Not relevant since embryos used in this study are sexually indifferent, while the ratio of female and male was expected to be approximately 1:1
Field-collected samples	The study does not involve samples collected from the filed.
Ethics oversight	Xenopus laevis were maintained and handled following the Guide for the Care and Use of Laboratory Animals of the National Institutes of Health. A protocol for animal use was approved by the Institutional Animal Care and Use Committee (IACUC) at Icahn School of Medicine at Mount Sinai.

Note that full information on the approval of the study protocol must also be provided in the manuscript.
